# The Geography of the Covid-19 Pandemic: A Data-Driven Approach to Exploring Geographical Driving Forces

**DOI:** 10.3390/ijerph18062803

**Published:** 2021-03-10

**Authors:** Frederik Seeup Hass, Jamal Jokar Arsanjani

**Affiliations:** Department of Planning, Geography and Surveying, Aalborg University Copenhagen, A.C. Meyers Vænge 15, 2450 Copenhagen, Denmark; frederiksh@plan.aau.dk

**Keywords:** machine learning, public health, Covid-19 pandemic, spatio-temporal analysis, spatial autocorrelation

## Abstract

The Covid-19 pandemic emerged and evolved so quickly that societies were not able to respond quickly enough, mainly due to the nature of the Covid-19 virus’ rate of spread and also the largely open societies that we live in. While we have been willingly moving towards open societies and reducing movement barriers, there is a need to be prepared for minimizing the openness of society on occasions such as large pandemics, which are low probability events with massive impacts. Certainly, similar to many phenomena, the Covid-19 pandemic has shown us its own geography presenting its emergence and evolving patterns as well as taking advantage of our geographical settings for escalating its spread. Hence, this study aims at presenting a data-driven approach for exploring the spatio-temporal patterns of the pandemic over a regional scale, i.e., Europe and a country scale, i.e., Denmark, and also what geographical variables potentially contribute to expediting its spread. We used official regional infection rates, points of interest, temperature and air pollution data for monitoring the pandemic’s spread across Europe and also applied geospatial methods such as spatial autocorrelation and space-time autocorrelation to extract relevant indicators that could explain the dynamics of the pandemic. Furthermore, we applied statistical methods, e.g., ordinary least squares, geographically weighted regression, as well as machine learning methods, e.g., random forest for exploring the potential correlation between the chosen underlying factors and the pandemic spread. Our findings indicate that population density, amenities such as cafes and bars, and pollution levels are the most influential explanatory variables while pollution levels can be explicitly used to monitor lockdown measures and infection rates at country level. The choice of data and methods used in this study along with the achieved results and presented discussions can empower health authorities and decision makers with an interactive decision support tool, which can be useful for imposing geographically varying lockdowns and protectives measures using historical data.

## 1. Introduction

According to the World Health Organization (WHO), there have been more than 79.2 million confirmed cases of Covid-19 globally and 1.7 million people are reported to have died hereof by the end of 2020 [1]. The Covid-19 pandemic has had and will continue to have life-changing effects on our lives around the world. Therefore, there is an urgent need for interdisciplinary studies to support health authorities and decisions makers with data-driven approaches for more effective recommendations and regulations for better management of such situations [2,3]. The Covid-19 outbreak has proven to be largely associated with geography, geographical information, and the geographical constellation of our cityscapes, land use information and transportation means, among others [4,5]. While epidemiologists and virologists and medics are trying to develop the right medicines, vaccines and medical solutions, an investigation of the geographical patterns of the pandemic and their relationship with geographical space with respect to the outbreak’s characteristics such as outbreak hot spots and cold spots, proximity of relevant urban elements, e.g., bars and restaurants, can help decision makers to impose more effective protective measures. These analyses help them to understand the nature and magnitude and severity of outbreak up to a certain timeframe and plan the future actions according to the historical development of the outbreak. Once such knowledge is gained, they can optimize daily societal activities more efficiently while lowering the infection risk. Thus, the aim of this study is to propose a data-driven approach for harvesting open geographic data (socio-economic data, biophysical data, mobility network, climatological data) released by open data initiatives and earth observation missions in order to (a) extract meaningful information about the geography of the pandemic such as hot- and cold spots in a temporal manner, i.e., weekly basis, that allow us to identify regions that have been constantly exposed to high infection rates or wise versa, (b) uncover the relationship between the outbreak and its geographical dimension. To do so, an application of statistical models and machine learning techniques will be tested allowing to explore what geographical settings support the outbreak. Finally, we will present appropriate open-source geospatial tools for achieving the aforementioned outputs.

The reminder of the paper is structured as follows: The introduction is followed by a literature review in Section 1.1. The data and methods used in the study will be presented in Section 2, while Section 3 presents our results. Section 4 presents discussions on our results and leads to conclusive remarks.

### 1.1. Related Work

Since the Covid-19 virus spread throughout the world causing a global pandemic, several studies have been conducted on the causes and factors that are essential to the spread of the virus. In this section, we present the state of the art on the studies focusing on geographic features and their correlation to the spread of virus together with machine learning techniques used in prediction spread and infection rates. The early studies related to the geography of Covid-19 focused on mapping the space and time of proven incidences and also to correlate these to geographic factors such as climate indicators and measures population density, population composition and human travel patterns. For instance, a study by Kim, Sun, and Castro [6] found a strong spatial autocorrelation and with the use of space-time scan identified clusters of Covid-19. Their findings proved that the disease initially spread in the densely and heavy populated capital region and over time moved to more distant regions of the country, furthermore single events of large spread and counter measures of these showed a space-time ripple effect in decreasing infection rates, which is also confirmed by the study of Giachino, et al. [7].

Investigating how climate indicators affect the spread, Briz-Redón and Serrano-Aroca [8] found no significant correlation between spring temperatures of Spain and Covid-19 cases, whereas Malki et al. [9] found Covid-19 was positively correlated to temperature and humidity, but also that these factors alone are not sufficient alone to predict infection rates. Briz-Redón and Serrano-Aroca [8] found that population density and number of visitors to a city had high correlations (*p* > 0.85) to infection rates, and Sannigrahi et al. [10] also found that total population, population income and poverty rates are strongly correlated to infection rates (*p* = 0.75, 0.68, 0.69 respectively). Mollalo, Behzad and Rivera [11] investigated demographic data across the United States and found that income inequality, household income, percentage of nurses and percentage of female black population, to be the most relevant explanatory demographic variables of countrywide infection numbers.

Studying the very first wave of Covid-19 in Wuhan China, Kraemer et al. [12] showed that by using positioning data of citizens, future infection hotspots can be accurately predicted. The study found an R^2^ = 0.89 between people travelling out of Wuhan and towards nearby cities and hereby causing spread of virus. Likewise, Kapoor et al. [13] was able to predict next-day cases within a single county in the United States with very high accuracy (RMSLE = 0.0109); this was done by plotting mobility data of people’s movement between counties on a graph neural network. It has, however, proven difficult for researchers to use geographic variables to predict the spread of virus through machine learning techniques. Wang et al. [14] were able to predict and forecast infection rates during the first wave of the pandemic in the spring of 2020 using a logistic growth model with a s-shaped curve, but they also found that the nature of the pandemic in the long term cannot be fitted to such a curve, and thus concluded that such a model can only poorly account for the fluctuations of infection seen over time. Besides being capable of predicting infection rates, studying them with machine learning techniques can provide knowledge about the importance and relationship between the included variables. Using machine learning, Gupta and Gharehgozli [15] showed that population density and pollution levels are closely correlated to virus spread.

Pollution levels and air-quality have indeed been seen to also change rapidly over the course of the pandemic, mainly due to the measures imposed on citizens’ ability to move and travel, and restrictions imposed on how businesses to run. Bao and Zhang [16] studied pollution levels across 44 Chinese cities, and found that across all pollution measures, the general Air Quality Index increased by 7.76% while the pollution indicators of SO_2_, PM_2.5_, PM_10_, NO_2_ and CO decreased by 6.76%, 5.93%, 13.66%, 24.67% and 4.58%, respectively. Collivignarelli et al. [17] similarly found that the early lockdown measures imposed in Milan (Italy) caused nitrogen dioxide and nitrogen oxide levels to fall by 61% and 74%, respectively, during the time of the lockdown.

Since the beginning of the Covid-19 pandemic, studies has been made to find factors that impact the spread of the virus, and we have presented some of the most relevant within the field of geographical analysis. Based on these, we aimed to implement novel techniques to map the spread of the virus within the European Union across space and time, examine explanatory variables and use them to predict infection rates. By including variables used in different studies presented, we can gain a deeper understanding of geographic driving factors in the Covid-19 pandemic. Within the geographic medium, our study additionally adds points of interest (POIs) data as a new set of variables that reflects the location of social hubs within urban areas. With the findings of the significant changes in pollution levels over the pandemic, these will also be included, allowing us to further investigate the correlation and explanatory factors between a large set of indicators for climate, pollution, population density, and POIs and the infection rate of Covid-19.

## 2. Data and Methods

### 2.1. Data

Covid-19 data was obtained from the European Centre for Disease Prevention and Control (ECDC), an agency under the European Union. The ECDC tracks and publish Covid-19 case numbers for the whole globe on a country level, within the European Union Covid-19 infection rates are also published at subnational regional levels. The dataset used is the *“Data on the weekly subnational 14-day notification rate of new COVID-19 cases”*, which contains weekly infection rates since week 13 (effectively week 14, since the data is based on bi-weekly reports) for each of the European subnational regions.

**Figure 1:**
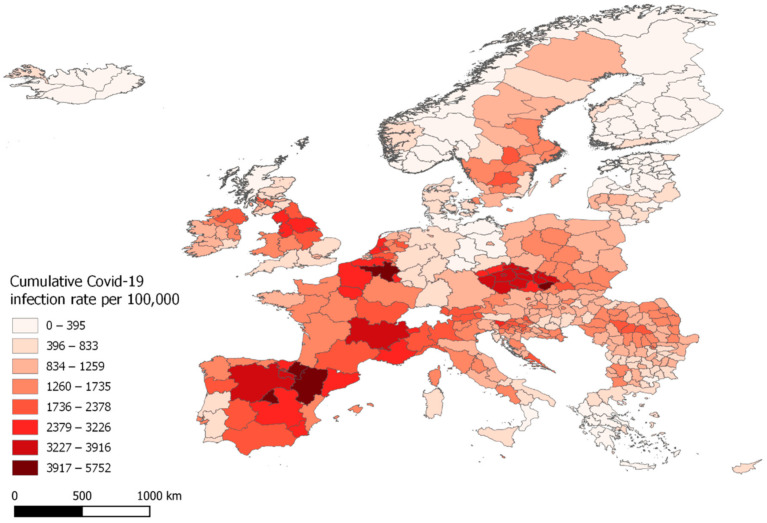
Cumulative Covid-19 infection rates per 100,000 inhabitant, from 23 March until 8 November 2020.

The subnational regions should be in accordance with the European Nomenclature of territorial units for statistics (NUTS) Regions, but it varies from country to country as per on what NUTS level the data is being recorded at. It has therefore been necessary to create a shapefile that consists of both NUTS 2 and NUTS 3 regions, and manually validate that each region matches the NUTS code provided by ECDC. This does, inevitably, results in countries being divided in an uneven amount of regions, with countries towards east such as Hungary, Estonia, Romania reporting cases at a municipal level, and the larger central European countries such as Germany, France, Spain reporting at a larger regional level. Cases reported in Wales UK, could not be identified at either NUTS 2 or 3 level, so the case data for Wales has been averaged in to a single region. The Covid-19 infection rate for each region are summed to a total infection rate covering the period of week 13 to week 45 (23 March 2020—8 November 2020), these can be seen in Figure 1.

POI data was gathered from OpenStreetMap (OSM), where all amenities for each country was collected through the overpass API and merged into a complete amenity point dataset. The number of amenities within each region were counted per capita by the 10 most abundant amenity types, i.e., restaurants, bars, cafés, gas stations, etc.

The amenities contained in OSM are crowdsourced data, which naturally raises the question of data quality. Since the amenity data is implemented as counts within the individual European regions, only the quality of completeness of the amenities are of concern. The quality of positional accuracy of points is not a cause for concern when working on a European scale. Unfortunately, completeness of amenities in the European countries was mostly studied a decade ago when the use and data contained in OSM was rising [18,19], and since then, most studies on accuracies of OSM amenities has been made with the focus of positional accuracy, and not completeness. A recent study on completeness of road and OSM’s POIs data in Europe found general high levels of quality in amenities and road data across major European cities [20]. The overall quality of OSM data in developed countries around the world is generally at a very high level [21], providing data in comparable quality to those of official data sources [22]. Places that has a high degree of tourism, i.e., most major European cities, also has a high completeness of the mapped amenities, whereas the data quality in rural areas remains to be tested [20].

Daily surface temperatures are available from Copernicus Climate Change Services (C3S) who monitor temperatures from ground stations across Europe. The daily mean temperature of 2019 was downloaded from the E-OBS database as a 0.1 degree regular gridded raster, and averaged in to an annual mean temperature. The annual mean temperature for each region is derived by using zonal statistics.

The Copernicus Atmosphere Monitoring Service (CAMS) monitors a set of variables related to air-quality and pollution levels across Europe, and distributes these as 0.1 degree rasters. Based on availability and documented cause for each pollutant, we chose “Non-methane VOCs” (volatile organic compounds), “Particulate matter < 2.5 µm (PM_2.5_)”, “Nitrogen dioxide” and “Particulate matter < 10 µm (PM_10_)” as indicators for air-quality and pollution levels. The four indicator data sets were downloaded as daily levels in the period of February through October for both 2019 and 2020 and the average year over year difference was calculated for each indicator and through zonal statistics transferred to the study regions.

The results is a data set covering 401 European regions with total Covid-19 infections a dependent variable (x0) and 17 independent geographic variables. All variables are listed in Table 1.

### 2.2. Methods

#### 2.2.1. Exploratory Data Analysis

Initially, an exploratory data analysis is carried out using the global Moran’s I statistics to determine spatial autocorrelation. The Moran’s statistic is a measure of how a variable of a region is correlated variable values of its surrounding regions, under the assumption that variable values are normally distributed across space. Moran’s I follows a normal distribution curve and tests whether clustering is appearing by calculating a z-score and *p*-values, which are measures for standard deviation values and level of significance. Moran’s I is a product of these values ranging from −1 to 1, where −1 is clustering of dissimilar values, 0 is perfect distribution of values and 1 is clustering of similar values and hereby showing spatial autocorrelation [23]. A predefined measure of neighbors are set, and each individual regions and its surrounding areas are tested for spatial autocorrelation against the complete data set. We used the *k* nearest neighbors to define neighborhoods, with differentiated k values for the two scales of the study. For the European spatial autocorrelation analysis we set *k* = 6 and for the Danish one we set *k* = 4.

The result of the analysis is grouped into four categories: hotspot, coldspot, doughnuts and diamonds. The hot- and coldspots are showing clusters of high infection rates and clusters of low infection rates, and the doughnuts and diamonds are showing regions with low infection rates surrounded by high infection rates and regions with high infection rates surrounded by low infection rates.

The data for the analysis are the total infection rates for each region gathered by accumulating the bi-weekly infection rates since week 14. The spatial autocorrelation is therefore a measure of clusters over the pandemic as a whole.

#### 2.2.2. Space Time Patterns

As an addition to the exploratory data analysis, an emerging hotspot analyses was made based on the results of spatial autocorrelation. The emerging hotspot analysis is a method that utilize a three-dimensional data set to locate clusters through both time and space. By doing a spatial autocorrelation analysis for each time slot of the available data (34 weeks) and adding these as layers on top of each other, it is possible to analyze how hot and coldspots change in space over time. The analysis was carried out as an extension to the spatial autocorrelation analysis done with the Pysal library in Python where we implemented space time patterns categorization inspired by the Esri tool for the same purpose [24]. The analysis look for 13 different scenarios for each region, these are seen in Table 2. The standard implementation of space time categories include an upper time interval threshold (t_max_) of 90% and a lower time interval threshold (t_min_) at opposite l0% in some of the categories. To better account for the changes in infection clustering in space it was decided to do multiple analysis with t_max_ and t_min_ at 75%, 25% and 55%, 45% for those categories.

The classifications are based on assessments of the results of spatial autocorrelation outputs stacked as layers through time, and these are then categorized by looking through the layers vertically. This is considered a simplified emerging hot spot analysis, as the analyses of the individual layers does include the layers above or below.

#### 2.2.3. Statistical Models: OLS & Geographically Weighted Regression

##### OLS

The ordinary least squares is a global regression model that seeks to investigate the relationship between a dependent variables (indicators) and a number of independent variables. The model is explained by the following equation [25]:

(1) $$y\;=\;\beta_{0}+B_{1}X_{1}+\beta_{2}X_{2}+\cdots \beta_{n}X_{n}+\varepsilon$$

where *y* is the dependent variable, in our case Covid-19 infection rates, *β* is the ordinary least squares estimated coefficients and *ε* is the residuals of the predicted values. The OLS is a global model since the equation assume the relationship between the dependent variable and the indicators to be constant for the whole data set. This is however a simplification of the investigated relationship, as this will often vary from location to location.

##### GWR

The geographically weighted regression (GWR) model is a similar regression model with the addition of adding geography to the equation by estimating local coefficients for local areas. GWR is thereby a local OLS model that varies over space. GWR is described by the OLS equation with the addition of coordinates for each location [25]:

(2) $$y\;=\;\beta_{0}\left(u_{i},v_{i}\right)+B_{1}\left(u_{i},v_{i}\right)X_{1}+\beta_{2}\left(u_{i},v_{i}\right)X_{2}+\cdots \beta_{n}\left(u_{i},v_{i}\right)X_{n}+\varepsilon$$

The local regression area is defined by a spatial kernel function and a bandwidth that can be defined as either distance (fixed bandwidth) or a neighborhood size (adaptive bandwidth). Only areas that are within the kernel are included in the GWR model. We used an adaptive bandwidth size defined by automatic model calibration and a Bi-square kernel.

GWR is capable of mapping how variable relationships vary across space and hereby to tell which variables best describes the variation seen in the dependent variable at different regions. We included all the variables seen in Table 1 in the GWR analysis. The model is evaluated by mapping local R^2^ values, variables coefficients and model residuals.

#### 2.2.4. Machine Learning Prediction

The geographic variables collected are a mix of static elements and dynamic climatic indicators. The population density, amenity counts and annual temperatures are elements that both reflects the geography and cityscapes, while at the same time, also tells something about the culture and local habits of the people living in a certain area. Meanwhile the air quality and pollution indicators are variables that change on the short term as a direct consequence of the level of activity and movement of people within a region. The year over year change should be a good indicator of how far from normal the activity level in a region has been, and thereby reflect the different lockdown policies implemented in the different countries and regions, a measure that elsewise is difficult to quantify.

By incorporating all these values in a prediction model, we aim at reflecting different aspects of geography, culture and level of activity in the regions across Europe. However, with lockdown measures being one of the key components in preventing the spread of virus, a pan European prediction model that does not directly include lockdown measures is very unlikely to outperform a model that does include such measures.

We therefore aim at carrying out the prediction at different levels of scale, with the broadest being all the 401 European regions where cases are reported. Narrowing down, the prediction is performed on the identified hot- and cold spots identified in the spatial autocorrelation analysis, reflecting the clusters of regions who did worst and best in terms of total Covid-19 infections. Lastly, the prediction is carried at a municipal level for the country Denmark, where the implemented policies have almost been uniform since the beginning of the pandemic.

We chose three different prediction models, all capable of predicting quantity values, and set out to predict the infection rate based on the 16 independent variables. The predictors of choice are random forest regressor [26], lasso regression [27] and support vector regression (SVR) [28,29]. The models were each chosen because they are widely used in similar prediction tasks.

## 3. Results

**Figure 2:**
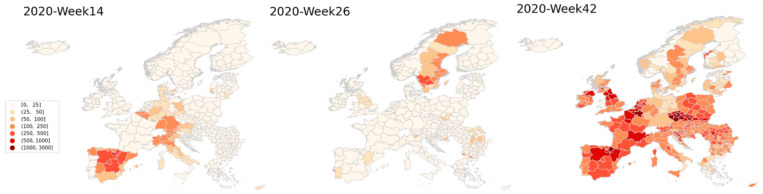
Bi-weekly infection rates at week 14, week 26 and week 42.

By November 2020, all European countries were affected by the Covid-19 pandemic, but the infection rates varied greatly between the individual countries and regions. Belgium, Spain and the Czech Republic are over the course of the whole period the countries with the highest infection rates. The regions of Wallone and Brussels in Belgium are the two worst hit, with total infection rates of 11,504 and 10,832 per 100,000 population. Iceland and Finland are the two countries with least infected regions. Five out of six regions in Iceland have less than 100 infected per capita and its capital region have had a total of 115 infected per 100,000 population. In Figure 2, bi-weekly infection rates for the included regions are visualized at three different weeks of 2020.

Over the whole time period of March to November, peak infection rates has occurred at different countries. In the spring of 2020 northern Italy and Spain were the worst affected, whereas in the summer Sweden was the only country with overall high infections, in the fall of 2020 the majority of countries were seeing surges in infections far higher than those observed in the spring.

### 3.1. Spatial Autocorrelation, EU and DK

The spatial autocorrelation analysis was carried out at the levels of all European Regions and for the municipalities in Denmark. The analysis covers a period of 32 weeks, from the start of April until start of November 2020, for which the infection rates has been accumulated.

**Figure 3:**
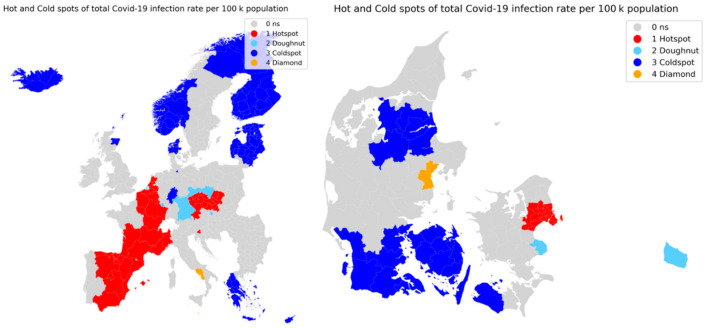
Hot and cold spots of total accumulated Covid-19 cases in the EU regions (left) and the municipalities of Denmark (right).

The spatial autocorrelation analysis seen in Figure 3 successfully identified clusters of hot- and coldspots together while also locating some doughnuts and diamonds. For Europe as a whole it is clear that the identified hotspots over the whole pandemic is the majority of Spain, France and Belgium and some parts of The Netherlands, the Czech Republic is also identified as a hotspot resulting in the surrounding regions of south Germany and Poland being doughnuts, i.e., low infection areas surrounded by high infected areas. Low infection areas, coldspots, are seen to mostly in the Nordic regions of Norway, Finland, the Baltic countries and Iceland, while Greece and Cyprus are the only southern regions reporting low infection rates.

For the municipalities of Denmark, high-infection areas are centered around the capital region of Copenhagen and its surrounding municipalities. High infection rates are also seen in the second largest city of Denmark, Aarhus, as this is identified as a diamond, i.e., having high infection rates while its surrounding municipalities have not seen similar high rates. The coldspot are seen to be the less populated areas of southern Denmark and central-north Jutland. The third largest city of Denmark, Odense in central Fyn, are within the large cold spot towards south and hereby not having significant higher infection rates than its surrounding municipalities.

As the spatial autocorrelation is based on accumulated infection rates, it does not show how infection rates has fluctuated over time in the individual regions, for this purpose we can assess the results of emerging hotspot analysis.

### 3.2. Space Time Patterns—Emerging Hotspot Analysis

The emerging hotspot analysis is a product of spatial autocorrelation over time, where each classification bin is a measure of how many times a regions has been either a hot- or coldspot or having no significance. As described in Section 2.2.2, two analyses are carried out each with a different threshold for how many times a region must be of a category that falls under one of the space time pattern categories.

#### 3.2.1. Emerging Hotspots—Europe

**Figure 4:**
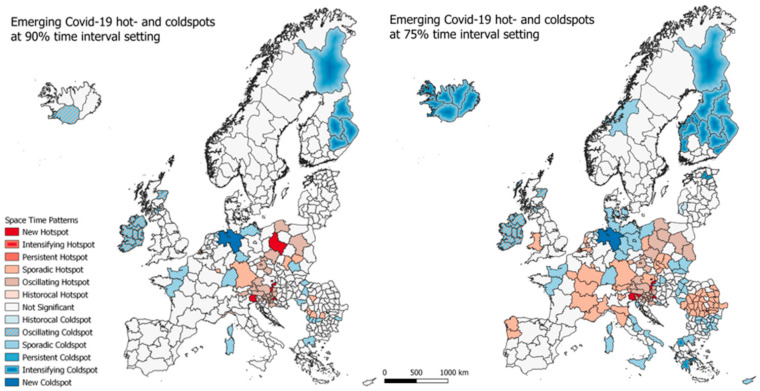
Emerging hotspot results for total cumulative infection rates from week 13 to week 45 in Europe at 90% (left) and 75% (right) time interval setting.

In Figure 4, the results of the emerging hotspot analysis for the EU regions are seen. Both at the 90% time interval setting and the 70% time interval setting, the majority of regions are seen to fall under the ‘not significant’ class, meaning that they did fall under any of the categories specified in Table 2. At the 90%-time interval map, new hotspots are seen to appear in northern Italy, parts of Austria (including Vienna), Croatia and the capital region of Poland. The sounding regions, that is mostly Austria and Croatia, are classified as oscillating hotspots, which are new hotspots that has in fact been a coldspot at an earlier time. Oscillating coldspots are centered on Ireland, Northern Ireland and some regions in Scotland, while some regions in Finland are seen to be intensifying coldspots which are regions that has generally have low infection rates and keep on doing so.

**Figure 5:**
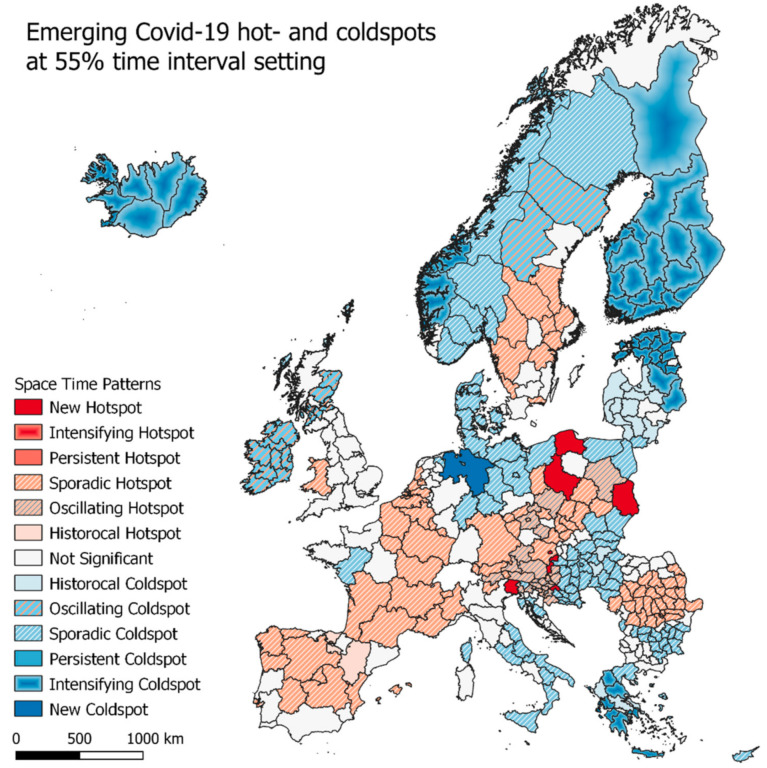
Emerging hotspot results for total cumulative infection rates from week 13 to week 45 at 55% time interval setting.

As seen in Figure 5, when lowering the time setting from 90% to 75%, more regions are expectedly classified in to the hot and coldspot categories. Especially the sporadic hot and coldspots are seen to be greater in number, with most of Romania, eastern France and northern Italy covered, and also Luxembourg, Wales and parts of The Netherlands (including its capital region) being sporadic hotspots. Sporadic coldspots are also seen to be greater in number, covering northern Germany, southern Denmark, eastern Hungary and Poland, and southern Italy. A larger part of Finland and all of Iceland is now classified as intensifying hotspots, and the regions of and around Ireland not changing their classification.

#### 3.2.2. Emerging Hotspots—Denmark

**Figure 6:**
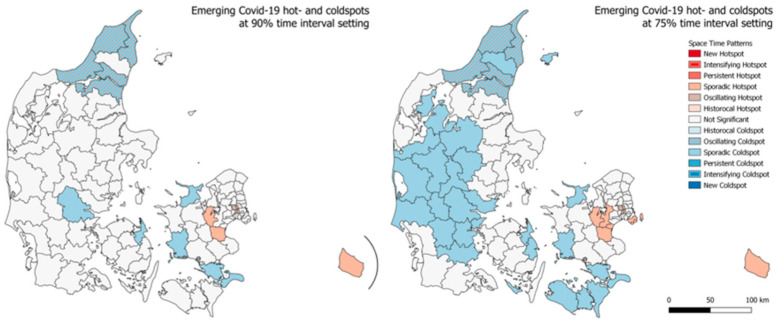
Emerging hotspot results for total cumulative infection rates from week 13 to week 45 in Denmark at 90% (left) and 75% (right) time interval setting.

**Figure 7:**
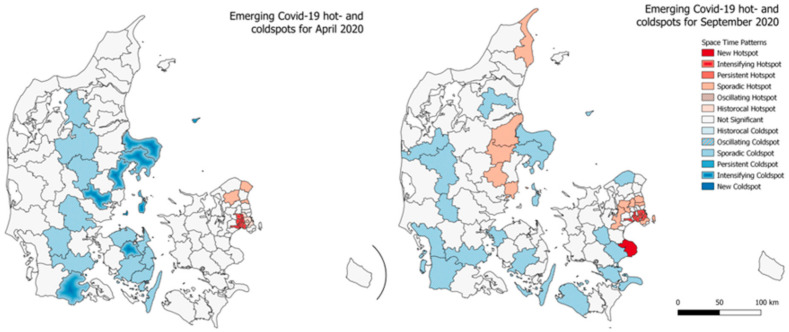
Emerging hotspot results for total cumulative infection rates in Denmark for April 2020 (left) and September 2020 (right) at 75% time interval setting.

**Figure 8:**
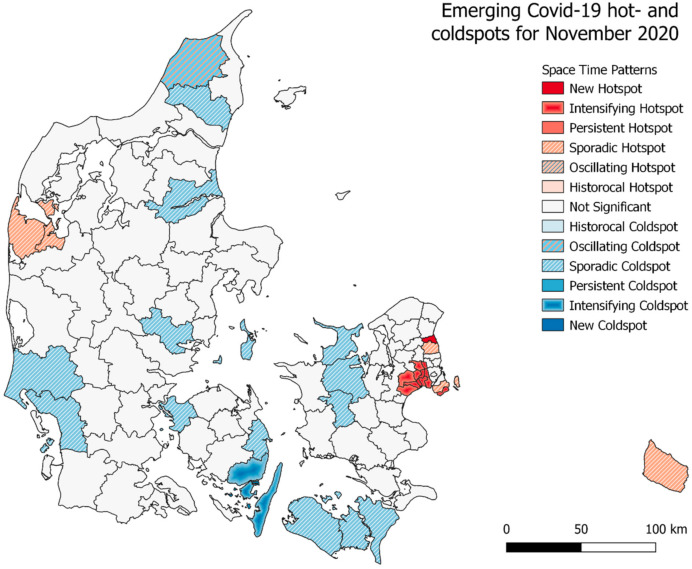
Emerging hotspot results for total cumulative infection rates in Denmark for November 2020 at 75% time interval setting.

A similar emerging hotspot analysis was carried out for the municipalities of Denmark, the results are seen in Figure 6. Interestingly, only the sporadic coldspots are seen to be greater in number when the time interval threshold is lowered from 90% to 75%. At the lower threshold, these sporadic coldspots covers large parts of mid- and western Jutland together with the municipalities towards south for the islands Lolland and Falster. For both the analysis, the outcome is otherwise roughly the same. In close proximity to Copenhagen, we see four oscillating hotspots and going a bit further away from the capital we see the sporadic hotspots. The oscillating coldspots are meanwhile seen to be at the opposite end of the country, in the very north of Jutland, where the 4th largest city of Denmark, Aalborg in also placed. Interestingly, neither the capital of the second and third largest cities of Denmark are categorized as any kind of hotspot. In Figure 7 and Figure 8, the emerging hotspot analysis is implemented on a monthly basis, showing in which parts of the country infection rates where increasing and decreasing at the given times.

### 3.3. Geographically Weighted Regression—EU

The GWR model was used to analyze how the 16 variables affected the total infection rate over the 401 European regions, the results consist of both the global regression model (OLS) and as local regression model. An automatic calibrated adaptive bi-square kernel [25] was used, with a bandwidth size of 104. See the global result statistics in Table 3 and local regression model results in Table 4.

Comparing the global regression model (OLS) with the local regression model (GWR) in Table 3, the residual sum of squares is seen to drop significantly from 219 to 66, and the AIC dropped from 931 to 752. The adjusted R^2^ value almost got a twofold increase from 0.432 to 0.765, indicating that the GWR model is able to account for 76.5% of the variation in Covid-19 infections. These results confirm the superiority of GWR in a case where we expect spatial variably in the relationship between the variables [30]. We can further investigate the statistics results for each of the variables included in the model, these are shown in Table 4.

**Figure 9:**
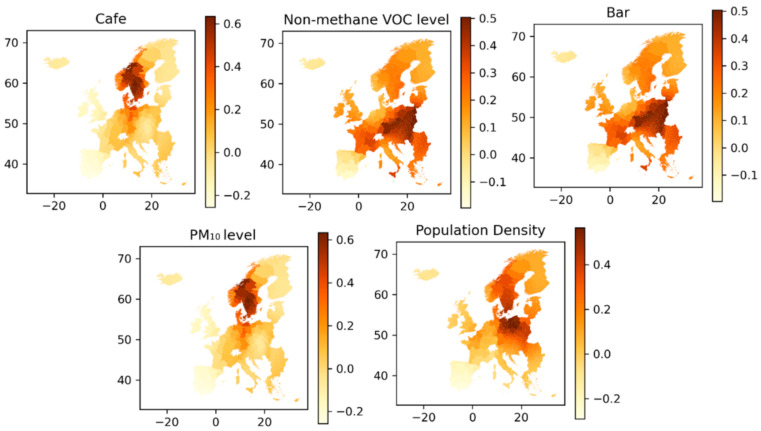
The coefficients of the 5 most significant variables in explaining total Covid-19 infections.

Inspecting the *p*-values of the individual variables in Table 4, the five variables having the greatest significance (*p*-value) on the dependent variable are X1 (Population Density), X3 (Cafes), X6 (Bars), X14 (Non-methane VOC) and X15 (PM_10_). The mapped coefficients for these five variables are shown in Figure 9. In the mapped coefficients, a similar pattern is seen between café and PM_10_ levels that the variables are of most influence in central Scandinavia, i.e., central Norway and Sweden, Denmark and Northern Germany. The other three variables do to some degree also share the same pattern, with the greatest influence on Covid-19 infection rates seen in eastern Europe, France, and to some degree Scandinavia and Italy.

**Figure 10:**
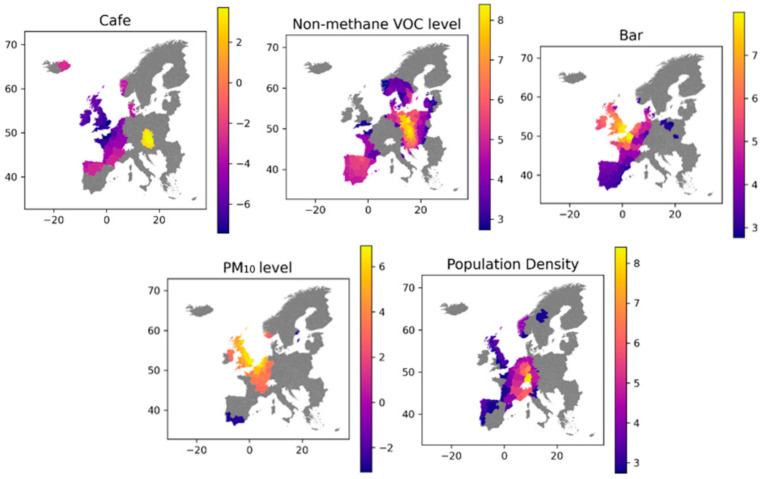
The significance of the 5 most significant variables in explaining total Covid-19 infections.

X14 (Non-methane VOC) and X15 (PM_10_) are both measures of air pollution, so these could be expected to have similar estimate coefficients, whereas the coefficients calculated in the OLS model in Table 4 show that X14 is positive and X15 is negative. The mapped coefficients in Figure 9 shows that the variable coefficients geographically are very different from each other, and similarly in Figure 10 where the two variables are seen to have significance in areas directly opposite to each other. Even though X14 and X15 are measures of air pollution, they each represent different emission sources and thus, do not follow the same geographic pattern

The charts in Figure 10 are showing in which regions each variable is having a significance towards the infection rate, this can both be a positive (high rates) and negative (low rates) impact on the rate. In the regions coloured in grey the variable did show any significance toward infection rates.

The population density is seen to have an overall positive effect on infection rates, with the greatest effect seen in the central European regions, covering Germany, France, Belgium, The Netherlands and England. Looking at cafes as bars, they seem to have effect in roughly the same area of western Europe, but interestingly each of these variables are affecting infection rates in opposite directions. Areas with a high number of bars per capita are having high infection rates, where the opposite is the case with cafes per capita in roughly the same areas. Since the two pollution level indicators, Non-methane VOC and PM_10_, are measured as year-over-year changes, higher values equal to a higher percentage yearly change. While we see the pollution indicators having impact at different geographic regions, the impact of them is highly positive. This indicates that regions that did not have a decrease in their pollution levels also had high Covid-19 infection numbers.

**Figure 11:**
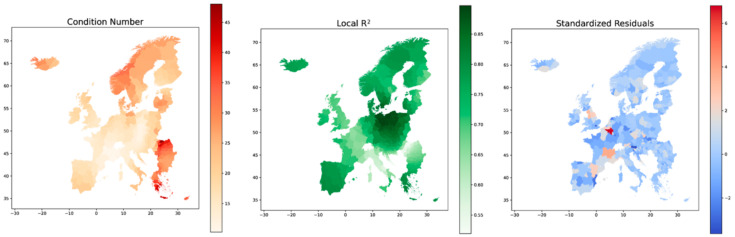
Geographic distribution of Condition Number, Local R^2^ values and Standardized Residuals for the GWR model of total Covid-19 infections.

Figure 11 shows the geographic distribution of the condition numbers, local R^2^ values, and standardized residuals. The condition number checks for redundancy in the variables used in the local regression task, and values above 30 are a cause for concern and results within these regions are not to be trusted [31]. For the most part, the condition numbers do not cause reasons for concern except at the very red regions of Romania and Greece. The levels here are in fact so high that the model output is not of any use in these regions. The R^2^ values are indicators of how well the model can fit the regression locally. The highest R^2^ values are in the regions around East Germany, Poland and Czech Republic, together with some parts of Scandinavia and Spain. The lowest R^2^ values are seen in Italy, parts of France and the North of Romania. Lastly the residuals are mapped to investigate if these appear to be clustered, to have a properly fitted model the residual should be evenly distributed in space. The residual map in Figure 11 does not indicate any spatial clustering of residuals.

### 3.4. Machine Learning Prediction

The machine learning prediction aimed at predicting the total infection rate for the included regions based on the variables described in Section 2.1 and Table 1. We tested three different models at four different scale levels, ranging from all of Europe, to selected regions of Europe and down to the individual municipalities of Denmark. All data included in the model are normalized and split into training and test data at a ratio of 70/30. The models are evaluated using the accuracy and mean absolute error (MAE) metrics, the accuracy should be as high as possible and MAE as low as possible. All models across all regions are ran over 10 iterations each and the evaluation scores are the average of the result of each iteration. The results of the models are seen in Table 5.

Across the included prediction models, the random forest is the best performing in both accuracy and MAE. The random forest prediction of all European regions is seen to have an accuracy of 52%, where, when only including the hot- and cold-spots, the same predictor is achieving 76% accuracy. The latter is of course a selection of the regions performing best and worst in terms of infection number, so a higher accuracy is expected here. However, the lowest MAE of all predictions is seen to be the pan European random forest prediction. The random forest prediction for the municipalities of Denmark showed 61% accuracy and a MAE of 0.095. This is finest level of scale for the predictions and also within a single country, so the policies for infection counter-measures are much more uniform across the regions.

**Figure 12:**
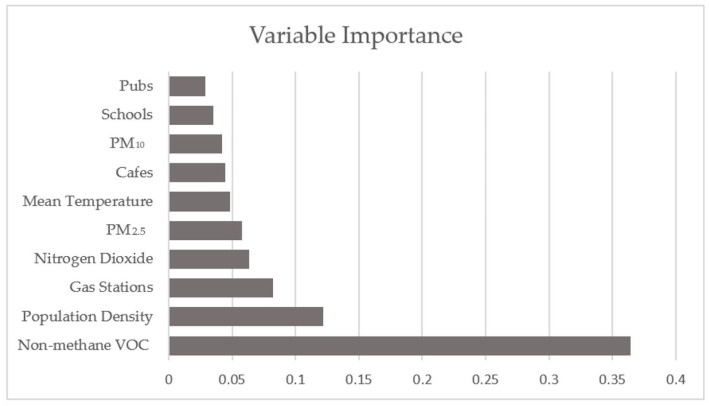
Variable Importance for Random Forest Regression of all EU regions.

The importance of the 10 most significant variables in the random forest pan Europe prediction is shown in the plot in Figure 12. It is clear that the year over year change of Non-methane VOCs are by far the most importance variable in this model, secondly comes population density, a variable that is also expected to be of significance importance to the spread of the virus. These variables are the only ones with a significance greater than 10%, and following them comes gas stations and other measures for air quality and pollution levels. This is according well to the results found in the geographic weighted regression models, where both Non-methane VOC and Population density showed great significance in explaining total infection rates.

## 4. Discussion

Throughout the paper, we have presented and implemented a number of tools that can be used to help understand how the spread of Covid-19 has evolved over time, where it is currently most active and what are the factors that affect this spread. For the most part of 2020 and continuing into 2021, Europe has been severely hit by the global pandemic with the first wave seen in the spring of 2020 followed by a summer with generally low infection rates and into the fall and winter of 2020 where the second, and in many cases, even more severe wave of infection raging over Europe. By the end of 2020, no country in Europe has remained safe and unaffected by the virus.

Our spatial autocorrelation analysis identified the central European regions as the hotspot of the total pandemic, and the more isolated and less populated Nordic regions as cold spots. This finding confirms our general understanding of how viruses spread, where densely populated and interconnected areas are at higher risk for a fast spread of pandemic [32,33]. As this analysis does not reflect the spread of virus over time, we used the finding of the spatial autocorrelation in an emerging hotspot analysis. This analysis is a powerful tool in mapping the pandemic in its current status and still factoring in how the individual regions have been affected in the past. The analysis can be implemented at many different scales of time and can hereby be used to study the spread of virus over long periods of time and down to monthly or even bi-weekly status of individual regions. The emerging hotspot maps are great tools for authorities to help deciding on where measures must be tightened or relieved while also serving as a great communication tool for the citizens to understand the basis of such decisions.

Our further analysis revolves around investigating what geographic factors affects the infection rate of Covid-19 in Europe, for this purpose we carried out a geographically weighted regression analysis. We found that the number of bars and cafes are significantly correlated to infection rates, confirming that places for social aggregation serve as infection source. Further analysis could be done at finer scales, to provide more in-depth knowledge of how the virus spread locally throughout cities and hereby help decision making regarding if such places should have restrictions imposed. Furthermore, we found that the air-quality and pollution indicators of Non-methane VOC and PM_10_ levels are also correlated with infection rates. This proves as an indication towards the level of activity in the society as lockdown measures expectedly have an effect on pollution levels [16], and indeed regions that had lower reduction in pollution levels have higher infection rates. Moreover, the GWR analysis showed a positive correlation between population density and infection rates, a finding that confirms the hypothesis that population density is associated with higher Covid-19 infection rates [15,34,35,36,37].

Finally, we implemented all the variables in a machine learning framework including random forest, support vector regression and lasso with the goal of assessing the predictability of infection rate solely based on the included variable features. Our pan European model was able to predict the infection rate with 52% accuracy and the single country model of Denmark resulted in 61% accuracy. A key component in predicting infection rates is naturally to which degree a country has introduced lockdown measures, a variable that is difficult to quantify and that was not introduced in the model. To achieve better prediction results, such a variable is highly recommended to include in the future works. The variable importance showed that pollution measures have a great importance in the prediction and given the fact that pollution levels and lockdown measures are closely correlated, we suggest that future research use pollution measures as one potential proxy of countrywide lockdowns. Certainly, while moving towards electric vehicles and renewable energies, such a proxy will not function. Actual levels of lockdown could also be quantified, but that is a challenging task since policies and their implementation are subject to human interpretation and behaviour. Due to the fact of dissimilar policies implemented, we found that implementing a single model across multiple countries is challenging, this is further concluded by result of the same model performing 10% better within a single country, i.e., Denmark, where policies are more uniform. This is largely due to a number of reasons such as (a) dissimilar number of COVID tests per capita rooted in the cost of Covid-19 tests from being free of charge (e.g., Denmark) or expensive (e.g., Greece, Romania), (b) not applying a universal standard for reporting infection numbers, (c) diversity of geodemographic distribution across each country, (d) dissimilar health services across Europe and, (e) wide diversity of cultures, behavioural factors, dominant background health issues in Europe.

## 5. Conclusions

This study presented a series of useful geospatial methods for studying the geography of Covid-19 and what factors facilitate the pandemic’s spread. We located European hot- and cold spots of infection rates and used this to further investigate and visualize how individual regions and municipalities have progressed over time. The emerging hot spot analysis is especially a powerful tool in understanding and visualizing where case numbers are rising and falling and hereby contribute to decision-making and raise understanding in the general public. We further examined the drivers of the pandemic through a pan European statistical GWR model and in a machine learning environment. The GWR model shows the most significant points of interest in explaining infection rates, while also showing that population density and air pollution are also strong explanatory factors. Our machine learning model, i.e., the random forest method, reached prediction accuracies of 52% and 61% for Europe and Denmark and confirmed that pollution data can be used as a relevant variable in predicting infections. In order to develop better prediction models in the future, we suggest including further variables that reflects lockdown levels of individual regions over time. Such a variable could be made from close examination of adopted policies, but could as well be derived from pollution levels.

## Figures and Tables

**Table 1:** Regions, Covid Infection rates and related variables.

Variable	Argument
Total infected [dependent variable]	X0
Population Density	X1
Restaurants per cap.	X2
Cafes per cap.	X3
Fast Food Places per cap.	X4
Pubs per cap.	X5
Bars per cap.	X6
Gas Stations per cap.	X7
Schools per cap.	X8
Doctors’ Offices per cap.	X9
Kinder gardens per cap.	X10
Annual mean temperature	X11
Nitrogen dioxide, year-over-year change	X12
Particulate matter < 2.5 µm (PM_2.5_, year-over-year change	X13
Non-methane VOCs (volatile organic compounds), year-over-year change	X14
Particulate matter < 10 µm (PM_10_), year-over-year change	X15

**Table 2:** Regions, Covid Infection rates and related variables (modified from [24]).

Space Time Category	Definition (t_max_ = 90%, t_min_ = 10%)
New Hotspot	Region identified as hotspot in the last time step that has not been a hotspot before
Sporadic Hotspot	Region identified as a hot spot at least once but in less than 10% of the time steps that also has not been a cold spot
Oscillating Hotspot	Region identified as hotspot in the last time step and been a hotspot in less than 90% of the time steps that also been a coldspot at least once
Historical Hotspot	Region that is not identified as hot spot in the last time step but has been a hot spot in more than 90% of the time steps
Intensifying Hotspot	Region identified as a hotspot in the last time step that has also been a hotspot in more than 90% of the time steps
Persistent Hotspot	Region identified as a hotspot in at least 90% of the time steps
New Coldspot	Region identified as a coldspot in the last time step that has not been a coldspot before
Sporadic Coldspot	Region identified as a coldspot at least once but in less than 10% of the time steps that also has not been a hotspot
Oscillating Coldspot	Region identified as a cold spot in the last time step and been a coldspot in less than 90% of the time steps that also been a hotspot at least once
Historical Coldspot	Region that is not identified as coldspot in the last time step but has been a coldspot in more than 90% of the time steps
Intensifying Coldspot	Region identified as a coldspot in the last time step that has also been a coldspot in more than 90% of the time steps
Persistent Coldspot	Region identified as a coldspot in at least 90% of the time steps
Not Significant	None of the above

**Table 3:** The global regression model (OLS) and the local regression model (GWR) results.

Model	Res Sum Squares	AIC	AICc	R^2^	Adjusted R^2^
OLS	219.230	927.850	931.448	0.453	0.432
GWR	66.922	654.585	752.719	0.833	0.765

**Table 4:** Variable statistics for OLS and GWR model. Est = Estimate, SE = Standard Error.

Variable	OLS		*p*-Value	GWR			
	Est	SE		STD	Min	Median	Max
X0	0.000	0.038	1.000	0.380	−0.909	−0.010	0.805
X1	0.111	0.043	0.009	0.137	−0.110	0.113	0.676
X2	0.109	0.063	0.084	0.222	−0.246	0.167	1.122
X3	−0.264	0.056	0.000	0.412	−1.612	−0.184	0.279
X4	0.007	0.049	0.893	0.306	−0.784	0.119	0.683
X5	−0.071	0.045	0.116	0.197	−0.761	−0.113	0.487
X6	0.195	0.054	0.000	0.359	−0.598	0.112	1.312
X7	0.011	0.051	0.828	0.441	−0.629	−0.018	1.487
X8	0.048	0.046	0.297	0.208	−0.568	−0.028	0.355
X9	−0.102	0.044	0.021	0.306	−1.074	−0.200	1.349
X10	−0.087	0.045	0.056	0.225	−0.610	0.010	0.780
X11	−0.134	0.052	0.010	0.322	−1.135	−0.280	0.640
X12	−0.115	0.049	0.019	0.353	−1.257	−0.022	0.794
X13	0.144	0.057	0.011	0.298	−0.997	0.039	0.500
X14	0.506	0.050	0.000	0.279	−0.124	0.372	0.956
X15	−0.142	0.050	0.004	0.376	−0.697	−0.069	1.360

**Table 5:** Machine Learning Prediction Results, MAE = Mean Absolute Error.

Area	Nr. of Regions	Model	Accuracy	MAE
Europe		Random Forest	**52%**	**0.079**
	401	Lasso	36%	0.153
		Support Vector Regression	51%	0.151
Europe—Hot- & Coldspots		Random Forest	**76%**	**0.099**
	128	Lasso	74%	0.285
		Support Vector Regression	73%	0.256
Europe—Hotspots		Random Forest	19%	**0.177**
	56	Lasso	- %	0.217
		Support Vector Regression	- %	0.221
Denmark		Random Forest	**61%**	**0.095**
	98	Lasso	35%	0.233
		Support Vector Regression	41%	0.232

Numbers marked in bold are the best achieved accuracy measures for the given area.

## Data Availability

All the data and codes used in this study are shared at https://github.com/jimjoker/Covid-19_geographic_analysis (accessed on 1 March 2021).
